# Abscisic Acid-Induced Autophagy Selectively via MAPK/JNK Signalling Pathway in Glioblastoma

**DOI:** 10.1007/s10571-020-00888-1

**Published:** 2020-06-23

**Authors:** Nan Zhou, Zixuan Wei, Zengxin Qi, Liang Chen

**Affiliations:** Department of Neurosurgery, Huashan Hospital, Fudan University, Middle Urumqi Road 12, Shanghai, 200040 China

**Keywords:** Abscisic acid, Autophagy, MAPK/JNK signalling pathway, Glioblastoma

## Abstract

As a widely known plant hormone, Abscisic acid plays an important role in the progress of planting cell and their stress response. Recently, we reported that ABA might play an anti-cancer role in glioma tissues. In the present study, the molecular mechanism of ABA anti-cancer was further explored in glioblastoma cells. By measuring LC3 puncta formation and conversion in glioblastoma cells, inhibiting the autophagic pathway, targeting the essential autophagic modulator beclin 1 with RNA interference, and analysing cellular morphology via transmission electron microscopy, we found that ABA-treated glioblastoma cells exhibited the features of autophagy. Specifically, ABA-induced autophagy in glioblastoma cells was mediated by the MAPK/JNK signalling pathway rather than the PI3K/AKT/mTOR axis. Moreover, the inhibition or knockdown of JNK specifically blocked ABA-induced autophagic cell death. ABA-induced autophagy was further confirmed in tumour-bearing mice and was accompanied by the inhibition of glioma growth in vivo. This report is the first to describe autophagy induced by ABA and mediated by the MAPK/JNK pathway in human cancer cells and tumour-bearing mice. These results may shed some light in new therapeutic strategies of glioma.

**Electronic supplementary material:**

The online version of this article (10.1007/s10571-020-00888-1) contains supplementary material, which is available to authorized users.

## Introduction

As a plant hormone, Abscisic acid (ABA) has been readily found in fruits and vegetables that can be naturally supplied by the dietary intake (Wasilewska et al. 2008). ABA plays a significant part in the progress of planting cell and their stress response and its medicinal applications have also recently attracted significant attention (Bassaganya-Riera et al. 2010; Li et al. 2011). Although the biosynthetic and metabolic pathways of ABA in animal cells are unknown, many animal and human cells have been shown to produce and release ABA. On account of a series of pioneering studies, Bruzzone et al. illustrated that ABA perform as a regulator between various cell functions, incorporate inflammatory processes in human granulocytes (Bruzzone et al. 2007), stem cell expansion (Scarfi et al. 2008), the activation of murine microglial cells (Bodrato et al. 2009), insulin release and glucose uptake (Bruzzone et al. 2012). Besides, ABA may also play a vital part in the stress response of mammals by downregulating the gene expression of the corticotrophin-releasing hormone, which is a central driver of the activity of the hypothalamic–pituitary–adrenal system (Qi et al. 2015). ABA Supplementation rescues high-fat diet-induced alterations in hippocampal inflammation (Ribes-Navarro et al. 2019). Recently, it was reported that ABA isolated from honey has antibacterial activity against Helicobacter pylori that links to the progress of the majority of peptic ulcers and several types of gastric cancers (Kim et al. 2017).

Malignant glioblastomas (GBMs) are the most common form of primary brain tumours and are associated with the highest mortality rate (Ostrom et al. 2014). The standard treatment for GBMs is the surgical resection of the tumour followed by concurrent radiation therapy (IR) and chemotherapy with temozolomide (TMZ). Unfortunately, advancements in recent decades have not significantly increased the overall survival of patients with this disease, and it remains an intractable problem to discover new therapeutic strategies to combat gliomas. Autophagy, a highly conserved cellular homeostatic process that can either suppress or promote tumours depending on the tumour type and stage is considered a new target for therapeutic interventions in brain tumours (Kaza et al. 2012). Interestingly, both TMZ and IR induce autophagy in glioma cells (Ito et al. 2005; Kanzawa et al. 2004), and pathological studies have shown that the levels of beclin 1 and microtubule-associated protein 1 light chain 3–2 (LC3-2), two biomarkers of autophagy, are lower in GBMs relative to lower-grade astrocytomas and normal brain tissue (Huang et al. 2010; Miracco et al. 2007). Furthermore, high beclin 1 and LC3 levels are associated with improved survival in GBM patients (Aoki et al. 2008; Pirtoli et al. 2009). However, the specific role of autophagy in promoting the survival or death of brain tumours in various therapeutic settings remains unclear.

Notably, Dr. Virginia Livingston (1976) proposed the use of ABA for cancer treatment four decades ago in an issued US patent (WC US3958025[P]). In our recent study, we reported that the ABA levels were twofold higher in low-grade gliomas than in high-grade gliomas. Remarkably, both cellular apoptosis and differentiation were augmented in the glioblastoma cells after ABA treatment. These results suggest that ABA may play an anti-cancer role in glioma by promoting cellular apoptosis and differentiation (Zhou et al. 2016). With respect to therapeutic strategies, glioblastoma cells are well known to be resistant to apoptotic stimuli, and their death occurs via autophagy (Kanzawa et al. 2004; Yao et al. 2003). In this study, we hypothesized that ABA may result in autophagic cell death in glioblastoma cells, making this compound a possible option for the progress of therapeutic drugs for glioma.

## Materials and Methods

### Antibodies and Reagents

The antibodies against beclin 1 (ab55878) and microtubule-associated protein 1 light chain 3 (LC3) (NB100-2220) were ordered from Novus Biologicals (LLC, USA). The antibodies recognizing 4E-BP1(#9644), phospho-4E-BP1(Thr37/46) (#2855), mTOR(#2972), phospho-mTOR (Ser2448)(#2971), p85S6K(#2708), phospho-p85S6K (Thr412) (#9234), p70S6K(#2708), and phospho-p70S6K (Thr389) (#9234) were obtained from Cell Signaling (Beverly, USA). The anti-GAPDH (KC-5G5) and anti-β-actin (KC-5A08) antibodies were obtained from Kangcheng (Shanghai, China). All other reagents were ordered from Sigma-Aldrich (St. Louis, USA) unless otherwise indicated.

### Cell Culture

The U87MG and A172 cell lines (Shanghai cell bank) were maintained in DMEM/F12 medium containing 10% foetal bovine serum (GIBCO, Grand Island, USA), 10 µg/ml streptomycin, and 10 units/ml penicillin in a 5% CO_2_ humidified atmosphere at 37 °C. U87MG cell line stably expressing LC3 was generated by the transfection of EGFP-LC3 plasmid using Lipofectamine 2000 transfection reagent (Invitrogen). Then cells stably transfected with EGFP-LC3 were selected in DMEM/F12 supplemented with 600 µg/ml G418 (Invitrogen) and 10% foetal bovine serum. The expression level of LC3 was measured by confocal microscopy.

### Immunofluorescence Staining

As previously described, U87, A172 cells were fixed with 4% paraformaldehyde in Tris-buffered saline at room temperature for 15 min. After three washes with TBS, samples were incubated with 0.3% hydrogen peroxide diluted in TBS for half an hour. Then samples were treated with 1% Triton X-100 for permeabilization followed by overnight incubation with anti-LC3 (1:300) antibody at 4 °C.

### Immunohistochemistry (IHC) and Periodic Acid Schiff (PAS) Stain

Tissues were fixed in 4% paraformaldehyde at 4 °C for 48 h, followed by embedding in paraffin and cut into 5 μm slices. The paraffin sections were gradient dehydrated in different concentrations of alcohol, and dewaxed in xylene, then the sections were incubated with 3% H2O2 and super-blocking reagent in room temperature for 10 min. The sections were subsequently incubated with the following primary antibodies at 4 °C. The next steps of immunohistochemistry stain were detected by ABsin Secondary antibody detection kit (ABsin, abs957) according to the manufacturer's protocols.

### Western Blot Analysis

Radioimmunoprecipitation assay (RIPA) buffer containing protease inhibitors (Roche, Indianapolis, USA) and 1 mM phenylmethylsulfonyl fluoride (Amresco, Solon, USA) was used to prepare cell lysates. Total proteins were separated on 12% SDS polyacrylamide gels followed by the staining with primary antibodies against LC3, beclin 1, mTOR, 4E-BP1, p85S6K, p70S6K, phospho-mTOR, phospho-4E-BP1, phospho-p85S6K, or phospho-p70S6K. GAPDH and β-actin were used for internal normalization. After the incubation with designated horseradish peroxidase-conjugated secondary antibodies, the bands were imaged using enhanced chemiluminescence (Amersham Bioscience, UK) and the band density was quantified using ImageJ software (NIH).

### RNA Interference

siRNAs targeting beclin 1, JNK, ERK or p38 (GenePharma, Shanghai, China) were transfected into cells to knockdown the target genes. In brief, cells (1 × 10^6^ cells/well) were grown in six-well plates overnight before the treatment with beclin 1 siRNA (5′-CCA CUC UGU GAG GAA UGC ACA GAU A -3′), JNK siRNA (5′-AAA GAA UGU CCU ACC UUC UTT-3′), ERK siRNA (5′-CAA GAG GAU UGA AGU AGA ATT-3′), p38 siRNA (5′-UGA AGA CUG UGA GCU GAA GTT-3′). The control siRNA sequences were: F, 5′-UUC UCC GAA CGU GUC ACG UTT-3′; R, 5′-ACG UGA CAC GUU CGG AGA ATT-3’.

Cells grown in 12-well plates were transfected with each siRNA duplex (1.5 lg) using RNAi-Mate (Gene Pharma). The medium was replaced with 1 ml fresh culture medium at 6 h post-transfection. After 24 h, cells were collected for RNA analyses.

### Transmission Electron Microscopy

Cell pellets were washed three times in PBS and then post-fixed in 1% osmium tetroxide for 1–1.5 h. After three rinses in PBS, samples were dehydrated in graded ethanol series and then transferred to acetone. Subsequently, cells were placed in a 50:50 mixture of acetone and Spurr’s epoxy resin for 60 min followed by overnight incubation with Spurr’s epoxy resin at room temperature. Then samples were cured in Beem capsules for 15 h at 70 °C and then sectioned into 100-nm-thick sections with a diamond knife on a Leica UC-7 instrument. The sections stained with uranyl acetate (2%) and lead citrate (0.2%) were photographed using a transmission electron microscope (Tecnai G2 Spirit BioTWIN, Hillsboro, USA). The de-identified samples were used to record autophagosomes in a minimum of 10 randomly chosen cells. The values reported here are the number of autophagic vacuoles/μm^2^/cell.

### Statistical Analysis

The statistical difference was evaluated using one-way ANOVA or two-tailed Student’s *t*-test, as appropriate, by SPSS software (SPSS Software, Chicago, IL, USA). A *p*-value < 0.05 was considered statistically significant. The data are expressed as mean ± SEM.

## Results

### ABA Actuated Autophagic Cell Death in Glioblastoma Cell Lines

**Fig. 1:**
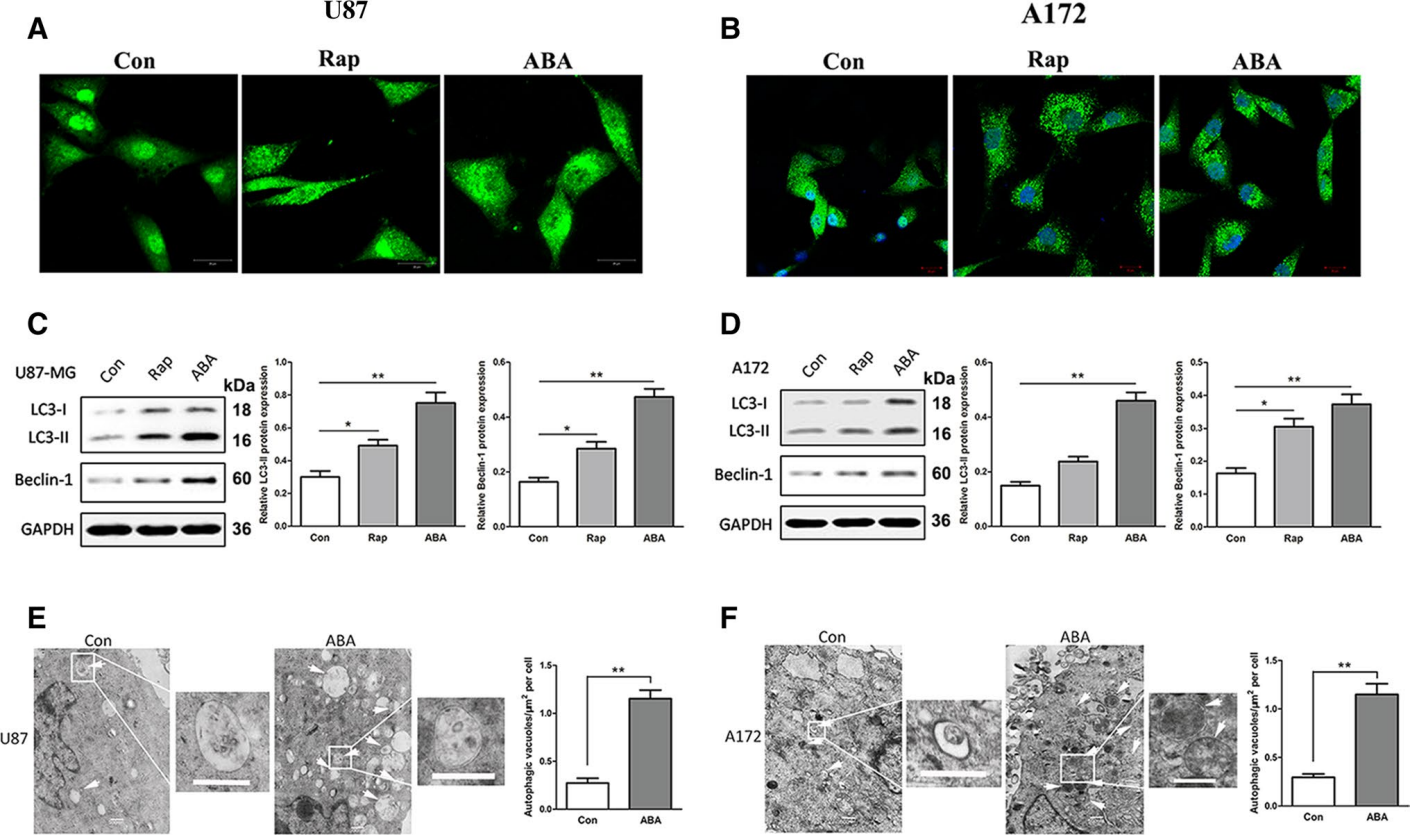
Abscisic acid inducing autophagy in glioblastoma cells. **a**, **b** Immunofluorescence with anti-LC3 antibodies showing endogenous LC3 puncta (green) in cells treated with DMSO (control), rapamycin (Rap 200 nM) and 200 μM abscisic acid (ABA) for 24 h; nuclei (blue) in U87 (**a**) and A172 cells (**b**) were visualized with DAPI. **c**, **d** Western blotting showing the endogenous conversion of LC3-I to LC3-II and the expression of beclin 1. Quantified by a densitometric analysis relative to GAPDH, the data are demonstrated on the right, *n* = 3. *P < 0.05 **P < 0.01 compared with controls. **e**, **f** Transmission electron microscopy (TEM) images of autophagic vacuoles in isolated cells. Autophagosomes are indicated with arrows. Regions within the boxes are magnified in the insets to indicate the double-membrane autophagosomes. Histogram values are presented as the mean ± S.E.M. in a minimum of 10 random cells. **P < 0.01 compared with controls. Scale bar, 500 nm

The level of the microtubule-related protein LC3, an autophagosome protein, was utilized as an indicator of autophagy. To survey the autophagy-inducing capacity of ABA, we initially observed the endogenous expression of LC3 in both U87 and A172 cells by laser confocal microscopy. Incubating the cells with ABA for 24 h increased the levels of LC3 puncta compared with controls (Fig. 1). Treatment with rapamycin, a commonly used autophagy inducer, also resulted in the extensive formation of LC3 puncta (Fig. 1a, b). We then tested the impact of ABA on the endogenous conversion of LC3-I to LC3-II and beclin 1 based on Western blotting. The expression levels of LC3-II and Beclin 1 have been expanded greatly in U87 and A172 cells in response to ABA treatment (Fig. 1c, d). Transmission electron microscopy (TEM) is considered the gold standard to assess autophagy. Therefore, we analysed the morphology of ABA-treated cells with TEM. Interestingly, we found that the ABA-treated glioblastoma cells exhibited features of autophagic cell death (Galluzzi et al. 2012). As shown in Fig. 1, after treatment with ABA, the dying cancer cells include intact nuclei, a growing number of autophagic vacuoles, and reduced numbers of cellular organelles (Fig. 1e, f).

### ABA Promoted Autophagy Flux by Enhancing Autophagosome Formation in Glioblastoma Cells

**Fig. 2:**
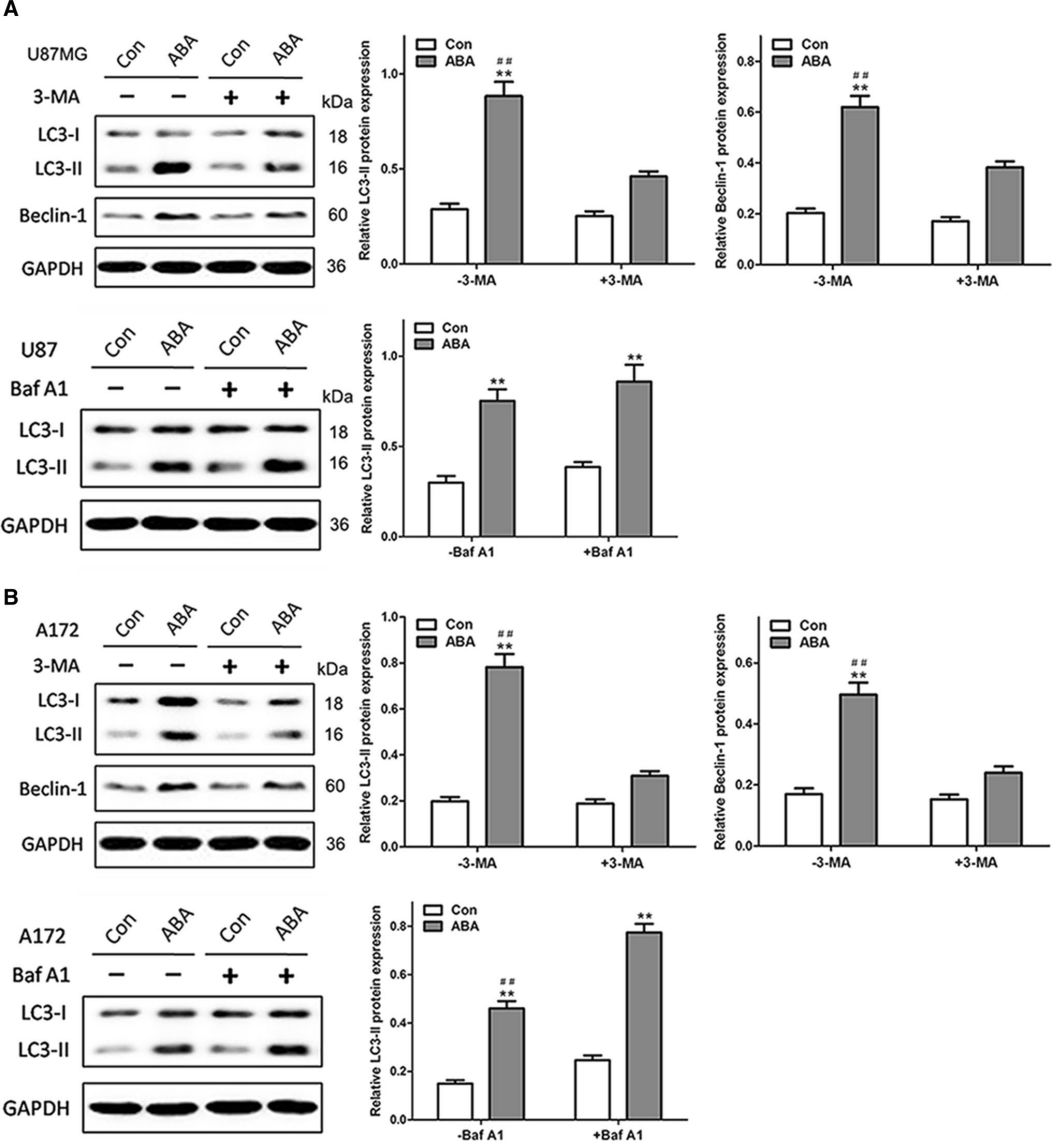
ABA inducing autophagic flux in glioblastoma cells. ABA-induced autophagy were reduced by the autophagy inhibitor 3-MA and added by enhancing autophagosome formation with bafilomycin A1 both in U87 cells (**a**) and A172 cells (**b**). **a**, **b** Western blotting at up row indicates the endogenous conversion of LC3-I to LC3-II and the expression of beclin 1 in U87 (**a**) and A172 cells (**b**) treated with DMSO (control) or ABA (200 μM) for 24 h in the presence and absence of 3-MA (10 mM). The data quantified by a densitometric analysis relative to GAPDH are shown on the right. **P < 0.01 compared with untreated controls, *n* = 3; ##*P* < 0.01 compared with 3-MA treatment, *n* = 3. Western blotting at low row indicates the conversion of LC3-I to LC3-II in U87 (**a**) and A172 cells (**b**) treated with DMSO (control) or ABA (200 μM) for 24 h in the presence and absence of bafilomycin A1 (Baf A1, 400 nM). The data quantified by a densitometric analysis relative to GAPDH are shown on the right. **P < 0.01 compared with untreated controls, *n* = 3; ##*P* < 0.01 compared with Baf A1 treatment, *n* = 3

Because ABA promoted autophagosome formation, we assessed the ability of 3-methyladenine (3-MA), a broadly utilized autophagy inhibitor, to attenuate ABA-induced autophagy. As demonstrated in Fig. 2, pre-treatment with 3-MA significantly attenuated ABA-induced increases in LC3-II and beclin 1 expression in U87 and A172 cells (Fig. 2a, b). To identify whether these changes were due to increases in autophagosome formation or decreases in autophagic degradation, bafilomycin A1 (Baf A1), which inhibits vacuolar H + ATPase and prevents fusion between autophagosomes and lysosomes, was utilized to assess autophagic flux. As shown in Fig. 2, ABA significantly increased the LC3-II levels in U87 (Fig. 2a) and A172 cells (Fig. 2b) in the presence of bafilomycin A1, strongly suggesting that ABA enhanced the formation of autophagosomes. These results also suggested that the autophagic flux induced by ABA was complete.

### ABA-Induced Autophagy Independent of the PI3K/AKT/mTOR Signalling Pathway in Glioblastoma Cells

**Fig. 3:**
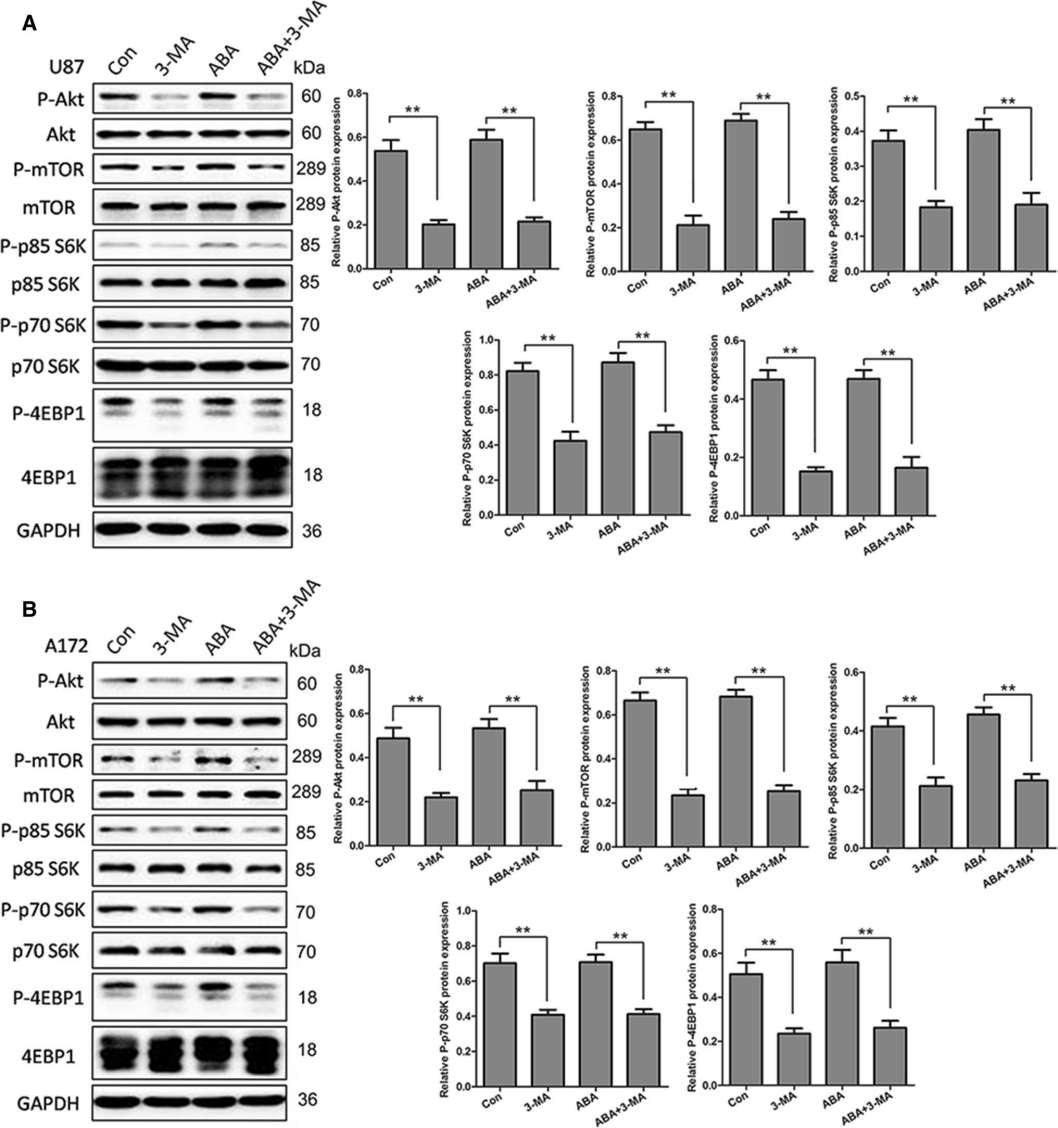
ABA inducing autophagy independent of the mTOR signal pathway. **a** U87 and **b** A172 cells treated with DMSO (control), 3-MA (10 mM) and ABA (200 μM) for 24 h were analysed by Western blotting to measure the levels of total and phospho-Akt, total and phospho-mTOR, total and phospho-p85S6k, p70S6K and 4E-BP1. The data quantified by a densitometric analysis relative to GAPDH are shown on the right. **P < 0.01, *n* = 3

We first investigated the participation of the PI3K/Akt/mTOR pathway, a well-known regulatory pathway in autophagy, in ABA-induced autophagy. The phosphorylation statuses of two well-characterized substrates of mTOR, ribosomal S6 protein kinase (p70S6K/p85S6K) and eukaryotic initiation factor 4E-binding protein 1 (4E-BP1), were used as indicators of mTOR activity. As shown in Fig. 3, unlike 3-MA, ABA did not reduce the phosphorylation levels of mTOR or its substrates, ribosomal S6 protein kinase (p70S6K/p85S6K) or eukaryotic initiation factor 4E-binding protein 1 (4E-BP1). We also analysed Akt signalling upstream of mTOR. Consistent with the observed mTOR activity, the phosphorylation status of Akt did not change in response to treatment with ABA (Fig. 3a, b). To further confirm the effect of ABA on mTOR activity, rapamycin was used as a positive control. As shown in supplementary Fig. 1, rapamycin almost completely blocked the expression of p-mTOR, p70S6K/p85S6K and 4E-BP1, whereas ABA did not affect mTOR activity (S-Fig. 1). These results indicate that ABA induced autophagy in an mTOR-independent method in U87 and A172 cell lines.

### ABA-Induced Autophagy via the MAPK/JNK Signalling Pathway in Glioblastoma Cells

**Fig. 4:**
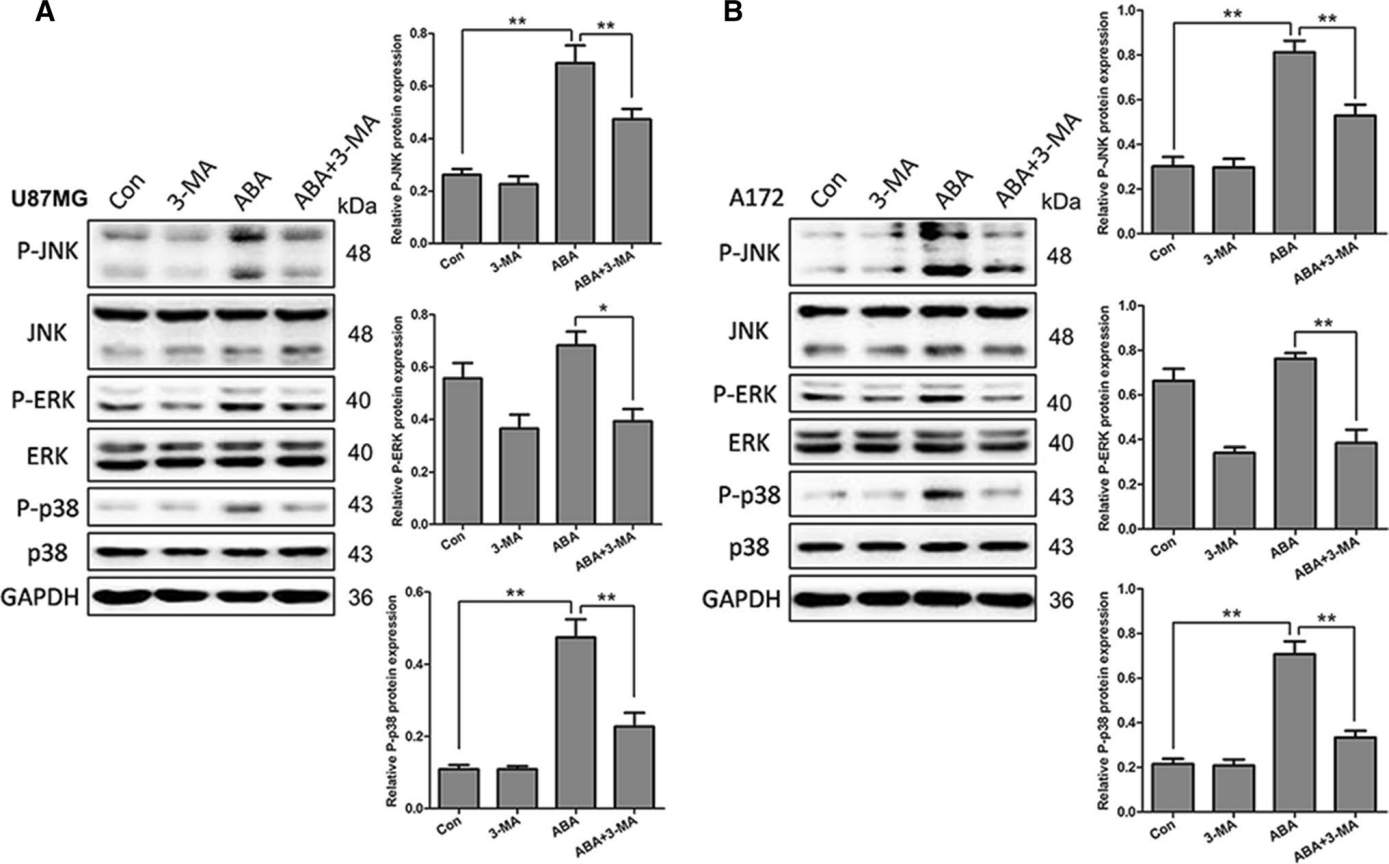
ABA inducing autophagy via the MAPK signalling pathway. **a** U87 and **b** A172 cells treated with DMSO (control), 3-MA (10 mM), ABA (200 μM) and ABA plus 3-MA for 24 h were analysed by Western blotting to measure the levels of total and phosphorylated JNK, ERK and p38. The data quantified by a densitometric analysis relative to GAPDH are shown on the right. *P < 0.05, *n* = 3; **P < 0.01, *n* = 3

MAPK signalling is another pathway that act an important part in autophagic cell death. To determine the association between ABA-induced autophagy and MAPK signalling, the effects of ABA on the phosphorylation of JNK, ERK, and p38 was examined in U87 and A172 cells by Western blotting. Interestingly, in U87 and Al72 cells, ABA treatment resulted in more than twice the expression of phosphorylated JNK and p38 compared with the control (Fig. 4a, b), but did not affect the expression of phosphorylated ERK (Fig. 4a, b). Remarkably, although the addition of the autophagy inhibitor 3-MA did not directly affect the expression of phosphorylated JNK and p38, pre-treatment with 3-MA obviously reduced the ABA-induced expand in p-JNK and p-p38 in U87 and A172 cells (Fig. 4a, b).

### Inhibition of MAPK/JNK Signalling Pathway Downregulated ABA-Induced Autophagy in Glioblastoma Cells

**Fig. 5:**
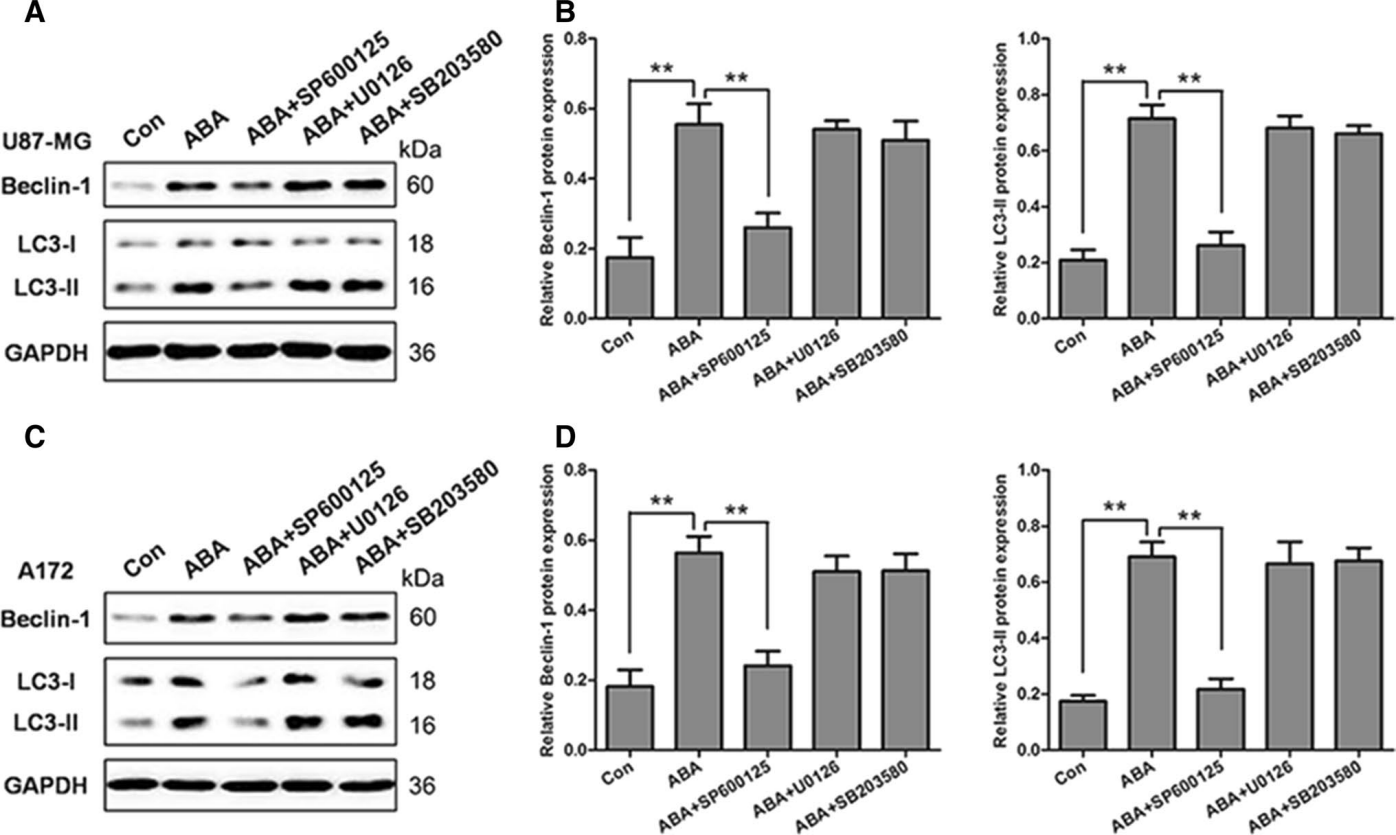
Causal relationship between the MAPK/JNK signalling pathway and ABA-induced autophagy. **a**, **b** U87 and **c**, **d** A172 cells treated with DMSO (control) or pre-treated with the JNK inhibitor SP600125 (25 μM), ERK inhibitor U0126 (20 μM) and p38 inhibitor SB203580 for 1 h, followed by treatment with ABA (200 μM) for 24 h were analysed by Western blotting to measure the levels of beclin 1 and LC3-II. The data quantified by a densitometric analysis relative to GAPDH are shown on the right. **P < 0.01, *n* = 3

To identify which MAPK signalling pathway that mediates ABA-induced autophagy, U87 and A172 cells were pre-treated with inhibitors of JNK (SP600125), ERK (U0126) and p38 (SB203580) for 1 h, followed by the treatment of 200 μM ABA for an additional 12 h. As a control, MAPK pathway inhibitors were added, which significantly reduced the increases in p-ERK, p-JNK, and p-p38 induced by ABA (S-Fig. 2). Interestingly, only the inhibition of JNK with SP600125 reduced the ABA-induced expression of LC3-II and beclin 1 to 50% and 60% of the control levels, respectively, in U87 and A172 cells (Fig. 5a–d). Conversely, inhibitors of ERK and p38 did not affect the expression of beclin 1 and LC3-II in either U87 or A172 cells (Fig. 5a–d). These data indicate that ABA induced autophagy specifically via the JNK signalling pathway in glioblastoma cells.

### Knockdown of Autophagy-Related Genes did not Affect the Expression of Key Proteins in the MAPK Signalling Pathway

**Fig. 6:**
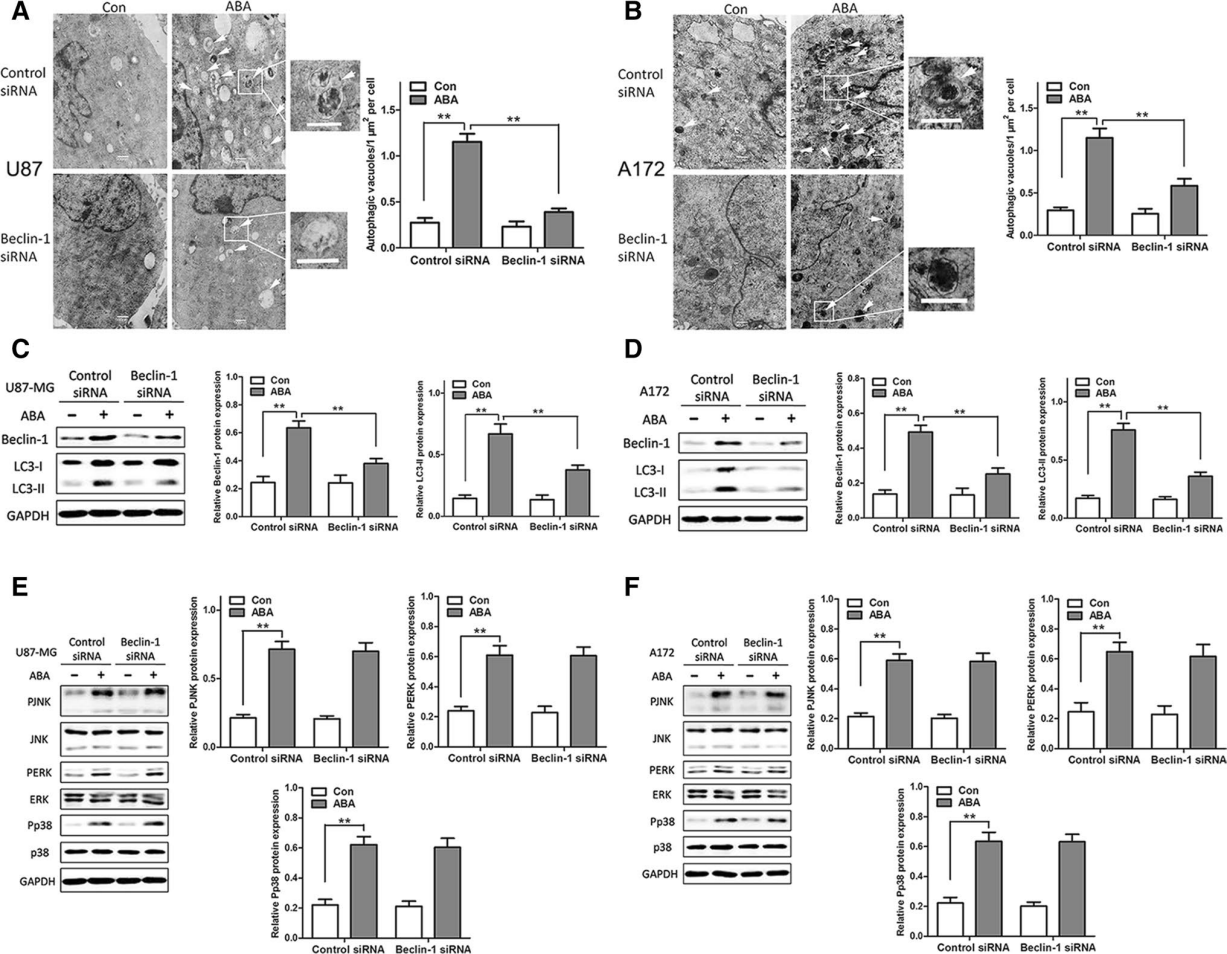
ABA inducing autophagy and activates the MAPK signalling pathway independent of beclin 1 RNA interference (siRNA) in glioblastoma cells. **a**, **b** Representative transmission electron microscope (TEM) images of autophagic vacuoles (arrows). Regions within the boxes are magnified in the insets indicating the double-membrane autophagosomes. Scale bars: 500 nm. Right panels in **a** and **b** show the quantification of ABA-induced autophagic vacuoles/μm^2^/cell with or without siRNA against beclin 1 in **a** U87 MG and **b** A172 cells. **c**–**f** ABA-induced expression of MAPK signalling pathway effectors independent of beclin 1 silencing in U87 (**c**, **e**) and A172 (**e**, **f**) cells measured by Western blotting. Values represent the mean ± SEM. **P < 0.01, *n* = 3

Beclin 1 is important for the signalling pathways participation in autophagosome formation. Depicted in Fig. 6a, b, transfection with small interfering RNAs (siRNAs) specific to beclin 1 markedly decreased the number of autophagic vacuoles in U87 and A172 cells, as revealed by TEM. Furthermore, the knockdown of beclin 1 significantly decreased the standard of endogenous beclin 1 and LC3-II proteins induced by ABA (Fig. 6c, d). However, the knockdown of beclin 1 did not affect the ABA-induced expression of phosphorylated JNK, ERK and p38 (Fig. 6e, f), indicating that the autophagy machinery acts downstream of the MAPK signalling pathway.

### Causal Relationship Between the MAPK/JNK Signal Pathway and ABA-Induced Autophagy

**Fig. 7:**
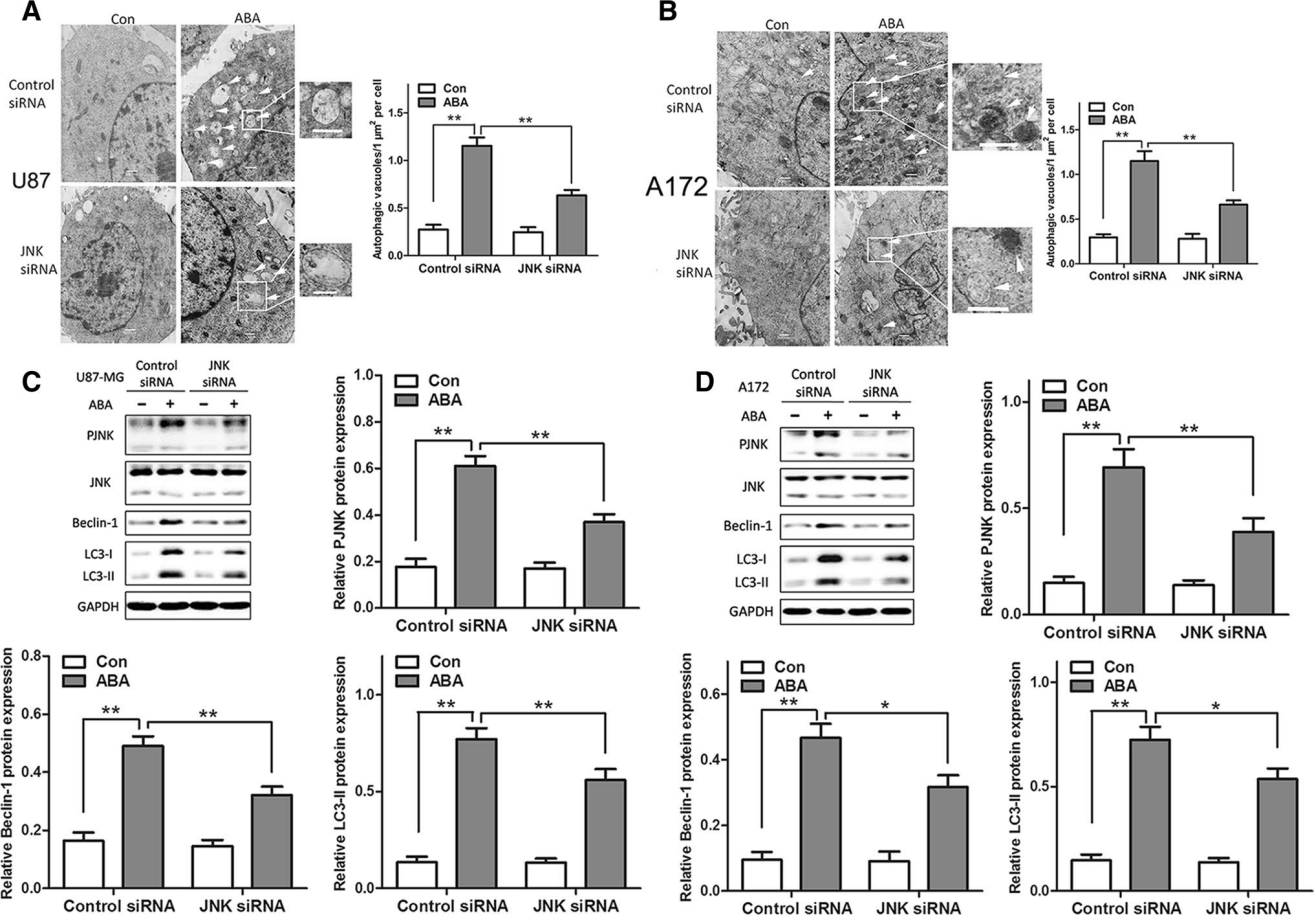
ABA inducing autophagy in glioblastoma cells independent of MAPK RNA interference (siRNA). **a**, **b** Representative transmission electron microscope (TEM) images of autophagic vacuoles (arrows). Regions within the boxes are magnified in the insets indicating the double-membrane autophagosomes. Scale bars: 500 nm. Right panels in **a** and **b** show the quantification of ABA-induced autophagic vacuoles/μm^2^/cell in U87 MG and A172 cells with or without siRNA. **c**, **d** ABA-induced expression of beclin 1 and LC3-II with or without JNK silencing in **c** U87 and **d** A172 cells as measured by Western blotting. Values represent the mean ± SEM; **P < 0.01, *n* = 3

To further characterize the causal relationship between the MAPK/JNK signalling pathway and ABA-induced autophagy, U87 cells and A172 cells were transfected with JNK, ERK and p38-specific siRNA or nonspecific siRNA. As shown in Fig. 7, JNK knockdown dramatically reduced the number of autophagic vacuoles induced by ABA in both U87 and A172 cells, as measured by TEM (Fig. 7a, b). Silencing JNK also significantly decreased the expression of beclin 1 and LC3-II in both U87 and A172 cells, as revealed by a Western blot (Fig. 7c, d). However, the knockdown of ERK or p38 neither affected the number of autophagic vacuoles nor the expression levels of beclin 1 and LC3-II in U87 and A172 cells (S-Figs. 3 and 4). These results suggest that the MAPK/JNK signalling pathway is causally related to ABA-induced autophagic cell death in glioblastoma cell lines.

### ABA Elevated Autophagic CELL DEATH and Inhibited Tumour Growth in Tumour-Bearing Mice

**Fig. 8:**
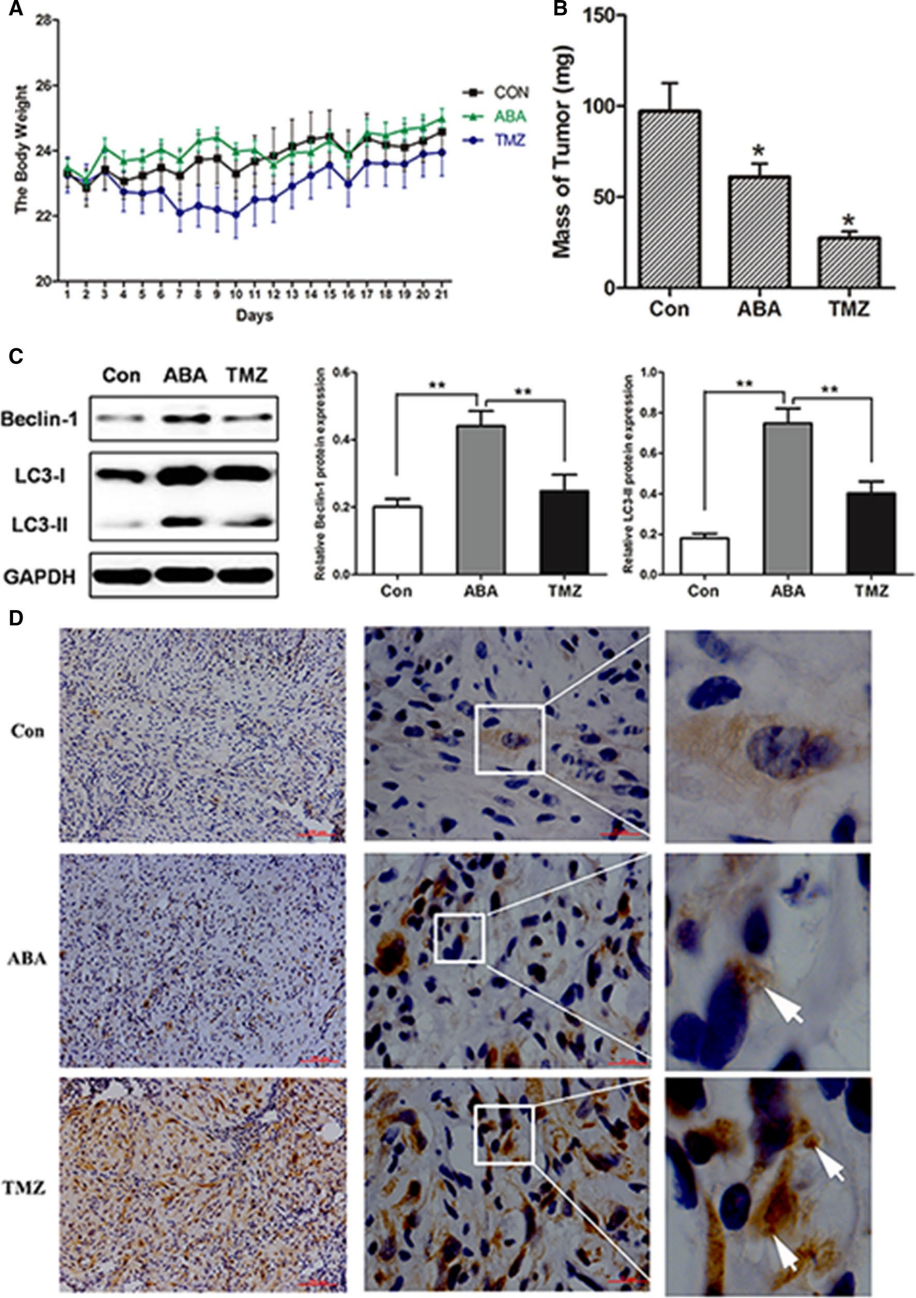
ABA inhibiting tumour growth in an allograft model of glioma. Balb/c nude mice were injected with U87 cells and then treated with DMSO (7 mice, negative control) or 15 mg/kg temozolomide (TMZ) (10 mice, positive control) and 60 mg/kg ABA (10 mice) once daily for 21 days. **a** The body weights of control, temozolomide- and ABA-treated mice over 21 days. **b** Tumours were collected after 21 days; *P < 0.05 compared with control. **c** Levels of beclin 1 and LC3-II autophagic tumour tissue from a tumour-bearing mouse model as analysed by Western blotting. The data quantified by a densitometric analysis relative to GAPDH are shown on the right; **P < 0.01, *n* = 3. **d** LC3 expression in tumour tissues as measured by immunocytochemistry. Regions within the boxes are magnified in the insets to indicate the puncta-like staining of LC3

On account of the ability of ABA to induce the autophagy in glioblastoma cells, then we tried the effects of ABA with tumour allografts in Balb/c mice. Specifically, Balb/c mice were subcutaneously injected with U87 cells. After 7 days the mice were given injections of control or ABA every day. Interestingly, body weight did not significantly differ between this two group, figuring that ABA treatment had a receivable safety range (Fig. 8a). Although ABA exhibited anti-tumour effects in tumour-bearing mice compared with controls, these effects were less than those of temozolomide. Moreover, ABA-treated mice exhibited an obvious diminution in tumour mass compared with the control group in three weeks (Fig. 8b). ABA-induced autophagy was further revealed by increases in the expression levels of LC3-II and beclin 1 in ABA-treated, tumour-bearing mice detected by Western blotting (Fig. 8c). Moreover, an immunohistochemical analysis further confirmed increases in LC3 expression in ABA-treated tumours, indicating the occurrence of autophagy (Fig. 8d). Thus, ABA effectively enhanced autophagy in vivo and was accompanied by inhibited tumour growth in a tumour-bearing animal model.

## Discussion

In the present study, the plant hormone ABA provoke autophagic cell death in glioblastoma cell lines via the JNK-MAPK signalling pathway. Furthermore, ABA inhibited tumour growth and enhances autophagy in an allograft model of glioma.

In plant, ABA has emerged as key players in the induction of autophagy under stress conditions. (Kulich and Zarsky 2014; Vanhee and Batoko 2011). Initially, we report that ABA induces autophagy in human cancer cell lines and in an animal model. Furthermore, by targeting the essential autophagic modulator beclin 1 with RNAi to inhibit autophagy and assessing the morphology of treated cells via EM, we found that ABA-treated glioblastoma cells exhibited features of either autophagic cell death as defined by the Nomenclature Committee on Cell Death (Galluzzi et al. 2012) or autophagy-associated cell death (AACD) as identified by Tasdemir et al. (Vicencio et al. 2008). These results are in accordance with the works before depicted high levels of autophagy induce cell death rather than supporting cell survival in glioma (Shchors et al. 2015; Shipman 2015). Among GBM patients, relatively higher levels of autophagy are related to preferable survival and prognosis (Aoki et al. 2008). Moreover, Chen et al. recently reported that the microRNA miR-129 is an effectively inducer of autophagy, and the intratumoural injection of miR-129 lentivirus significantly constrained tumour growth (Chen et al. 2016). ABA-induced autophagic cell death is of interest as a therapeutic strategy for GBM because the stimulation of autophagy is considered a potential therapeutic approach. However, information regarding the use of autophagy-enhancing agents in clinical trials for the treatment of glioblastoma is limited. Specifically, mammalian target of rapamycin inhibitors are approved by the US Food and Drug Administration (FDA) for cancer therapy (Huang and Fingar 2014), and the combination of mTOR inhibitors with TMZ during radiotherapy to trigger autophagic cell death is thought to be a promising treatment approach (Koukourakis et al. 2016). A phase II study of temsirolimus, an analogue of the allosteric mTOR inhibitor rapamycin, showed that this agent was clinically effective in 30% patients treated for recurrent glioblastoma (Galanis et al. 2005). Hainsworth et al. administered BVZ with everolimus, another inhibitor of mTOR, after radiochemotherapy to 68 glioblastoma patients; the results showed that this regimen improved the progression-free survival of patients (Hainsworth et al. 2012). However, another phase II study of one hundred patients did not distinguish any useful impact on survival in patients with glioblastoma (Ma et al. 2015). Interestingly, Shipiman et al. recently showed that the imipramine and the ticlopidine synergistically induced autophagy and slow down glioma progression in mice (Shchors et al. 2015; Shipman 2015).

To elucidate the molecular mechanisms underlying ABA-induced autophagy, we studied possible signalling pathways in glioblastoma cells. In contrast to a previous study, in which the PI3K/AKT/mTOR signalling pathway was shown to be involved in anti-cancer agent-induced autophagy in glioma (Sami and Karsy 2013), we found that ABA-induced autophagy in glioblastoma cells is independent of the PI3K/AKT/mTOR signalling pathway. Instead, we discovered that the MAPK/JNK signalling pathway is essential for ABA-induced autophagy. The discrepancies between these findings may be due to differences in the cancer type and doses used in the different experimental models. The MAPK/JNK pathway was recently reported to potentially regulate autophagy in glioma (Zhou et al. 2015) and was also reported to control the balance of apoptosis and autophagy in response to chemotherapeutic agents. Specifically, the JNK signal transduction pathway plays a crucial part in autophagic cell death and defence mechanisms. Liu et al. showed that palmitate promotes autophagy and apoptosis through ROS-dependent JNK and p38 MAPK. Here, we discovered that ABA promoted the expression of phosphorylated JNK, ERK and p38, but only the knockdown of JNK blocked ABA-induced autophagy, and neither ERK nor p38 knockdown affected ABA-induced autophagy. These outcomes were in line with the results of the studies before showing that autophagy is regulated by the JNK pathway. Moreover, arsenic trioxide, evodiamine, and the downregulation of protein kinase CK2 can reportedly induce autophagic cell death by modulating the MAPK signalling pathway in human glioblastoma cells (Chiu et al. 2011; Kanzawa et al. 2005; Liu et al. 2013; Olsen et al. 2012).

In conclusion, this report is the first to show that treatment with the phytohormone ABA results in autophagic cell death in human cancer cells and tumour-bearing mice and that this effect is mediated by the MAPK/JNK pathway. These results may provide a promising avenue for the development of glioma therapies.

## Electronic supplementary material

Below is the link to the electronic supplementary material.

Supplementary file1 (TIFF 966 kb)

Supplementary file2 (TIFF 681 kb)

Supplementary file3 (TIFF 9469 kb)

Supplementary file4 (TIFF 9095 kb)

Supplementary file5 (DOCX 15 kb)
