# Supplementary figures and images for: Abscisic Acid-Induced Autophagy Selectively via MAPK/JNK Signalling Pathway in Glioblastoma
Source: Cell Mol Neurobiol. 2020 Jun 23;41(4):813–26. doi: 10.1007/s10571-020-00888-1 (PMC7997842; doi:10.1007/s10571-020-00888-1)

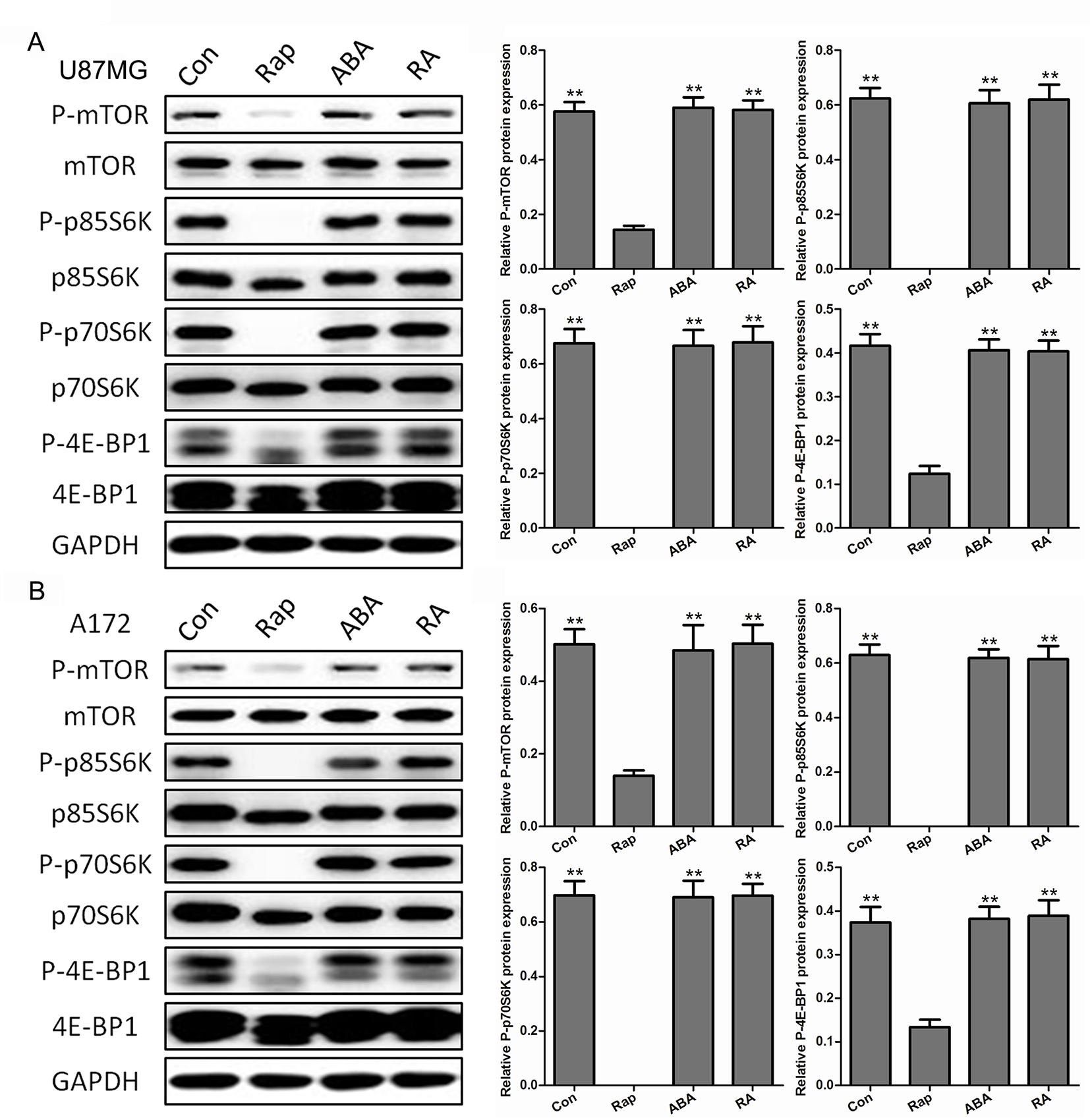

Supplement: Supplementary file 1 — Supplementary file1 (TIFF 966 kb) [file 10571_2020_888_MOESM1_ESM.tiff]

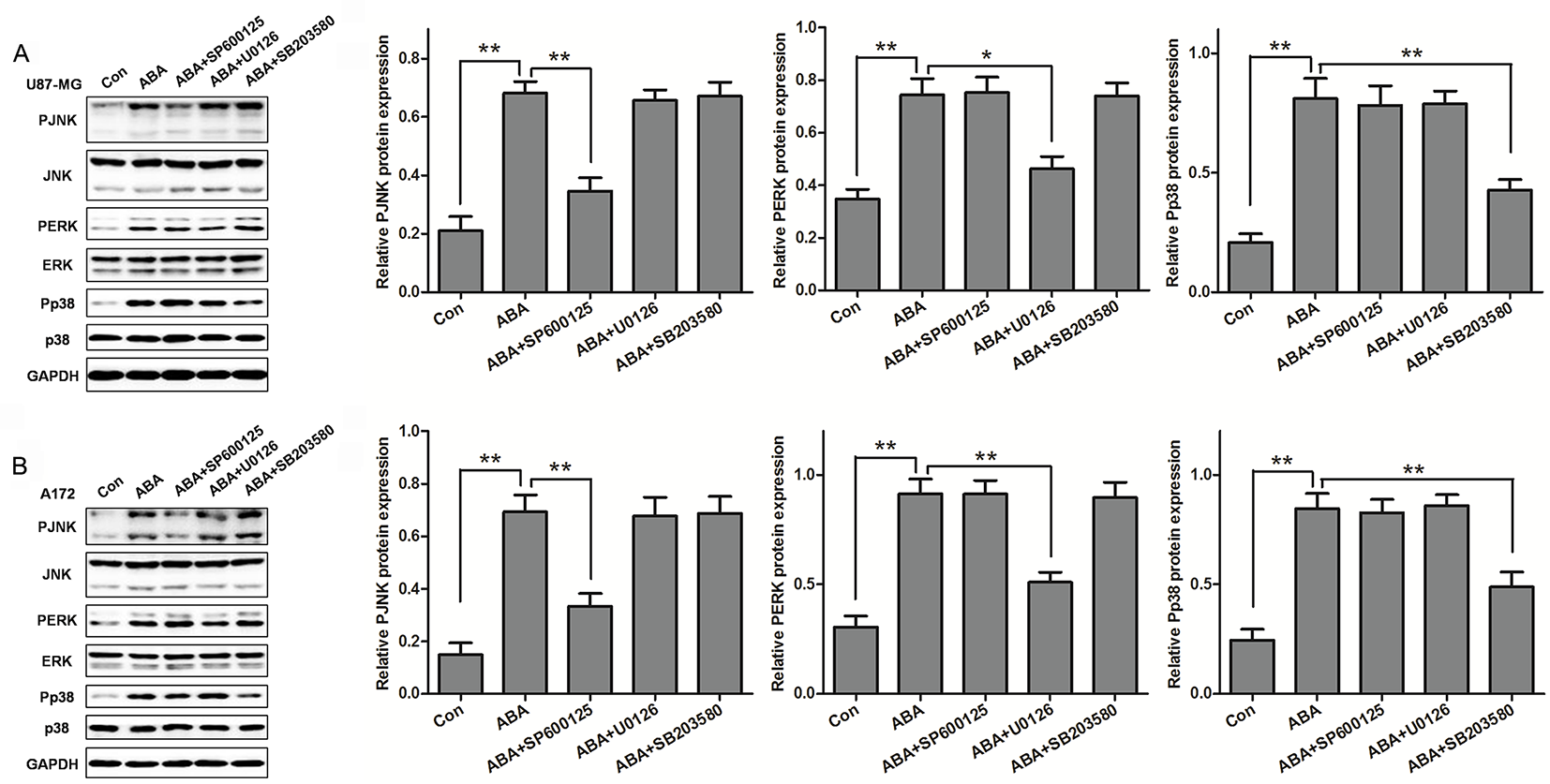

Supplement: Supplementary file 2 — Supplementary file2 (TIFF 681 kb) [file 10571_2020_888_MOESM2_ESM.tiff]

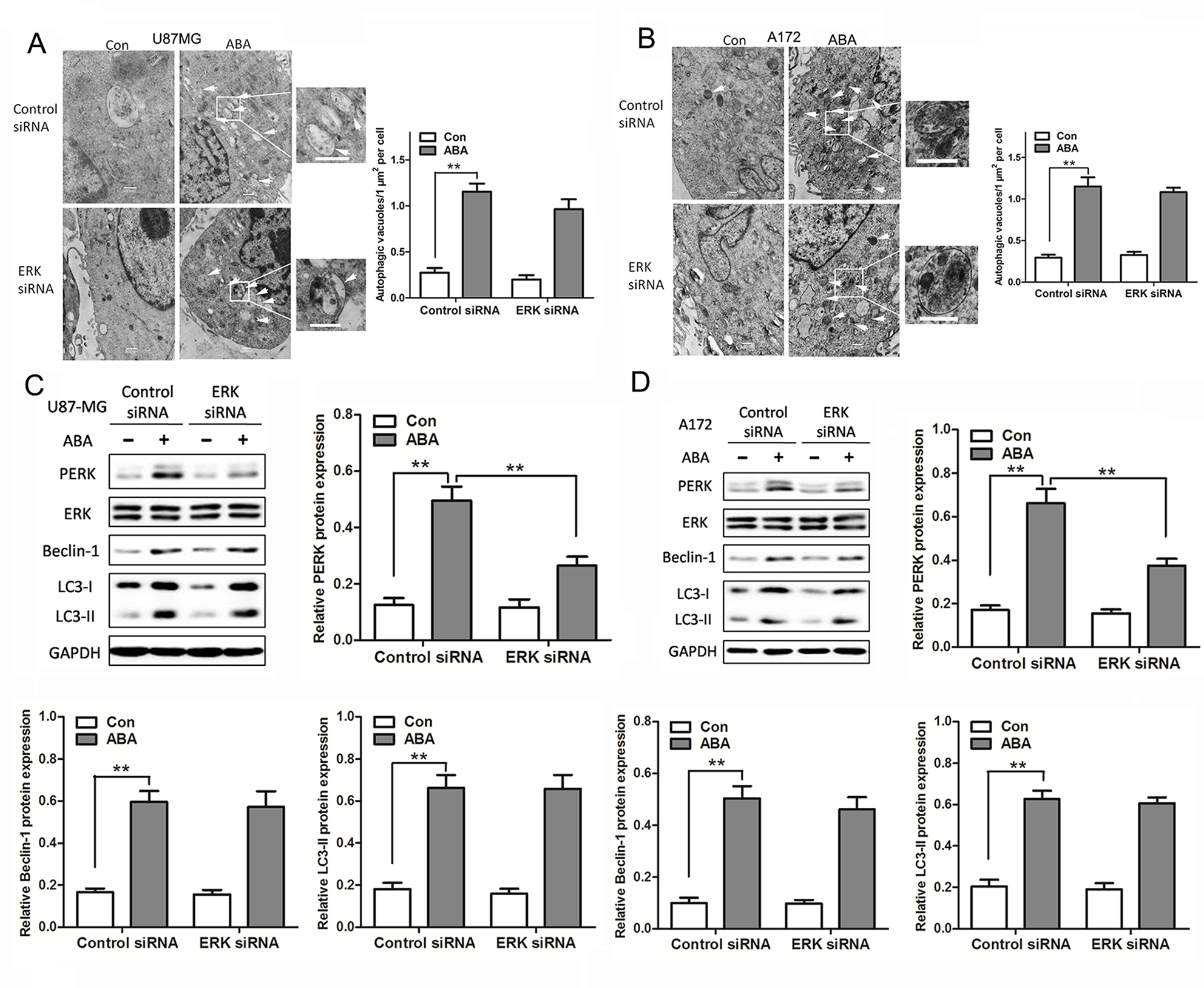

Supplement: Supplementary file 3 — Supplementary file3 (TIFF 9469 kb) [file 10571_2020_888_MOESM3_ESM.tiff]

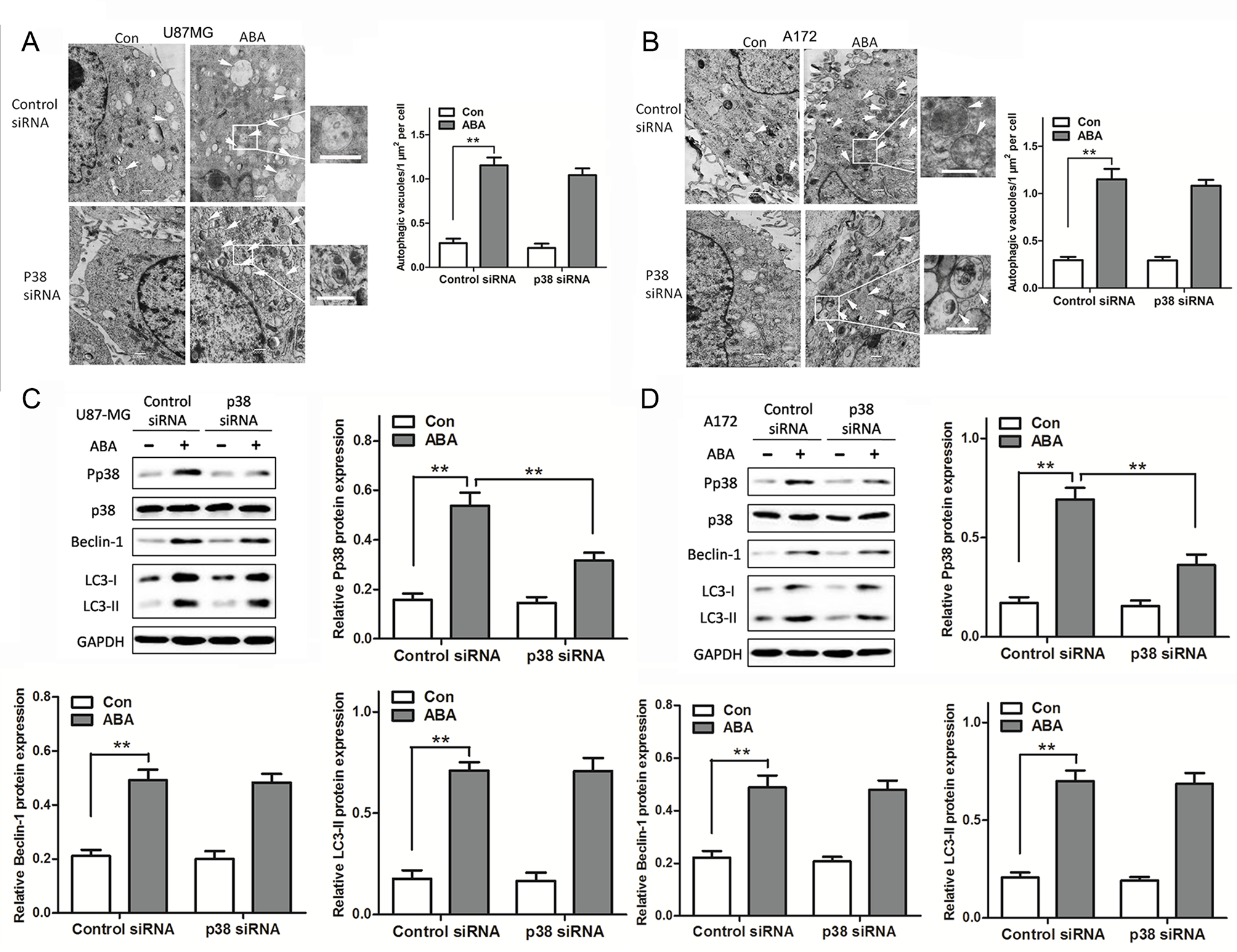

Supplement: Supplementary file 4 — Supplementary file4 (TIFF 9095 kb) [file 10571_2020_888_MOESM4_ESM.tiff]
