# Supplementary material for: Abscisic Acid-Induced Autophagy Selectively via MAPK/JNK Signalling Pathway in Glioblastoma
Source: Cell Mol Neurobiol. 2020 Jun 23;41(4):813–26. doi: 10.1007/s10571-020-00888-1 (PMC7997842; doi:10.1007/s10571-020-00888-1)
Supplement: Supplementary file 5 — Supplementary file5 (DOCX 15 kb) [file 10571_2020_888_MOESM5_ESM.docx]

**Supplementary Figure Legends**

**Supplementary Figure 1** ABA had no effect on the expressin of mTOR pathway activity with the treatment of rapamycin.

As shown in figure, mTOR inhibitor of rapamycin almost completely blocked the the expression of p-mTOR, p70S6K/p85S6K, 4E-BP1 , whereas ABA showed again no effect on the mTOR activity. U87MG (A) and A172 (B) cells treated with DMSO (control), rapamycin (Rapa 200 nM) , ABA (200 μm) as well as retinoid acid (RA), a positive control, for 24 h, were analyzed by western blotting for levels of total- and phospho- p-mTOR, p70S6K/ p85S6K, 4E-BP1. Quantified data by densitometric analysis relative to GAPDH are shown on the right (*: P<0.05, n=3, **: p < 0.01, n = 3).

**Supplementary Figure 2** Positive control of MAPK pathway inhibitors.

U87MG (A) and A172 (B) cells treated with DMSO (control), pretreatment of JNK inhibitor SP600125 (25 um), ERK inhibitor U0126 (20 um) as well as p38 inhibitor SB203580 for 1 h, then treated with ABA (200 μm) for 24 h, were analyzed by western blotting for levels of total- and phospho- JNK, ERK and p38. Quantified data by densitometric analysis relative to GAPDH are shown on the right (**p < 0.01, n = 3).

**Supplementary Figure 3** ABA induced autophagy in glioblastoma cells with or without RNA interference (siRNA) of MAPK/ERK signal pathway.

(A-B) Representative transmission electron microscope (TEM) images of autophagic vacuole (in arrows). Regions within the boxes are magnified in the insets indicating the double membrane autophagosomes. Scale bars: 500 nm. Right panel in (A) and (B) shown the quantification of ABA induced the number of autophagic vacuoles /μm2 per cell in U87 MG and A172 cells with or without siRNA of ERK. (C-D) ABA induced expression of Beclin-1 and LC3-II with or without silencing ERK in U87MG (C) and A172 (D) cells measured by western blot. Values represented: mean±SEM; **P < 0.01, n=3.

**Supplementary Figure 4** ABA induced autophagy in glioblastoma cells with or without RNA interference (siRNA) of MAPK/p38 signal pathway.

(A-B) Representative transmission electron microscope (TEM) images of autophagic vacuole (in arrows). Regions within the boxes are magnified in the insets indicating the double membrane autophagosomes. Scale bars: 500 nm. Right panel in (A) and (B) shown the quantification of ABA induced the number of autophagic vacuoles /μm2 per cell in U87 MG and A172 cells with or without siRNA of p38. (C-D) ABA induced expression of Beclin-1 and LC3-II with or without silencing p38 in U87MG (C) and A172 (D) cells measured by western blot. Values represented: mean±SEM; **P < 0.01, n=3.
